# GENETIC AND SEROLOGICAL BIOMARKERS AS PREDICTORS OF PARKINSON’S DISEASE COGNITIVE IMPAIRMENT: A SYSTEMATIC REVIEW

**DOI:** 10.1093/geroni/igae098.3989

**Published:** 2024-12-31

**Authors:** Hajir Mohammed, Mohamed Atif Mohamed Yousif, Mohamed Elhassan, Yassein Elhussein, Dina Badawi, Sarra Gabralla, Nadir Abdelrahman

**Affiliations:** Tele-Geriatric Research Fellowship, East Lansing, Michigan, United States; Tele-Geriatric Research Fellowship, East Lansing, Michigan, United States; Royal College of Surgeons Ireland, Waterford, Dublin, Ireland; Dubai Medical College for Girls (DMCG)), Dubai, United Arab Emirates; University of Texas Health (UTHealth) Houston, Texas, United States; Tele-Geriatric Research Fellowship, East Lansing, Michigan, United States; Michigan State University, East Lansing, Michigan, United States

## Abstract

Parkinson’s disease (PD) is the second most common neurodegenerative disorder in the elderly population. Cognitive impairment (CI) is one of the most common and important non-motor symptoms in Parkinson’s disease and the identification of predictive biomarkers for it would help in its management. Databases were searched systematically down to Jan 2019. Selection criteria focused on studies with serological and genetic biomarkers. Other radiographically or clinically related papers were excluded. Total of 9 studies with 11 different biomarkers were assessed and found to be significantly associated with cognitive decline in PD. These include APOE4 allele mentioned in 2 studies (RR= 2.98), (RR= 7.4). miR-203a-3p/miR-16-5p ratio (AUC= 0.7593), Serum high mobility group box-1 levels (RR= 1.398), ganglion cell inner plexiform layer complex in the macula thickness (RR= 0.553), B12 (RR= 0.793), CSF NfL (HR= 2.82). MIA, CRP and albumin group (HR= 4.3), Neuroexosomal EAAT-2 (P= 0.003), and P16 (P= 0.003). Biomarkers can serve as potential predictive tools. Their integration into routine assessments could enhance the stratification of patients at risk for cognitive impairment, allowing for tailored therapeutic approaches and preventive strategies aimed at mitigating cognitive decline. However, they warrant further investigation to elucidate their roles in the pathophysiology of cognitive impairment in PD. Longitudinal studies are needed to validate these biomarkers and determine their utility in predicting cognitive decline over time. Additionally, exploring the interplay between these biomarkers and other neurobiological factors may uncover novel therapeutic targets.

